# Fig extract drying: The relationship between the main operating parameters of a pilot‐scale spray dryer and product specifications

**DOI:** 10.1002/fsn3.558

**Published:** 2017-12-06

**Authors:** Maryam Kalantari, Mehrdad Niakousari, Soroush Haghighi‐Manesh, Mehrdad Rasouli

**Affiliations:** ^1^ Department of Food Science and Technology Islamic Azad University, Varamin‐ Pishva Branch Varamin Iran; ^2^ Department of Food Science and Technology School of Agriculture Shiraz University Shiraz Iran; ^3^ Department of Food Science and Technology School of Agriculture Tarbiat Modares University Tehran Iran

**Keywords:** fig, Formulation, Glass transition temperature, Powder characterization, Spray drying

## Abstract

This study aims to optimize extraction and drying conditions of fig syrup. Drying was done in a pilot scale two‐fluid nozzle spray dryer. A total of 27 experiments were conducted with varying inlet air temperatures, air flow rates, and also a maltodextrin (MD)‐ low methoxyl pectin (LMP) ratios. While feed rate, feed temperature, and compressed air flow rate of the atomizer were kept constant. The results of *differential scanning calorimetry* revealed that high levels of glucose and fructose in the extract resulted in a low glass transition temperature of fig syrup. By an increase in the inlet air temperature, the powder bulk density decreased. However, the MD:LMP ratio and the air flow rate were not significantly effective (*p < *0/05) in changing the bulk density. SEM micrographs of spray‐dried particles and particle size distribution analysis showed that particles were largely in a range of 5 to 50 μm. The best powders were obtained at an inlet air temperature of 170°C.

## 1 INTRODUCTION

Formulating industrial food products is an unfathomable issue in food sciences which are considered to have both nutritional and functional characteristics of food ingredients. The findings of this class of food industry can also be applicable in the field of pharmaceutical and cosmetic industries because of today's diligent consumers who prefer to use natural and organic products. Food engineers aim to manipulate natural food ingredients for achieving great functional properties in food industries, guaranteeing the long‐term preservation as well as maintaining nutritional values to provide health promotion aspects. Therefore, reaching these objectives of natural plant sources are always the most appropriate option. In formulating healthy food products, the fig fruit is an excellent source. According to the USDA report, fig is rich in vitamins, minerals, and dietary fiber, and also contains high amounts of natural antioxidant compounds which can prevent lipoprotein oxidation in plasma and reduce the risk of getting cancer and cardiovascular disease. Furthermore, high fructose and glucose content of fig make it suitable for formulation of foods with natural sweetener (Chang, Alasalvar, & Shahidi, 2016; Foster, 2013; Wojdyło, Nowicka, Carbonell‐Barrachina, & Hernández, 2016).

Because of the economic reasons, damaged and deformed figs are more applicable in industry as they can be converted to fig extract and fig powder. So it is vital to select those extraction conditions which may not cause heat damage to nutrients, and also to inhibit temperature‐dependent adverse reactions such as the Maillard reaction (Barbosa‐ Cánovas & Juliano, 2005; de Oliveira, Coimbra, de Oliveira, Zuniga, & Rojas, 2016). The main challenge of fig syrup extraction is to provide an appropriate gentle process. Based on documented assays, there are few reports concerning the production process of fig extract. Meanwhile Farahnaky, Ansari, & Majzoobi (2010); Razavi, Pourfarzad, Sourky, & Jahromy (2010); Ansari, Farahnaky, Majzoobi, & Badii (2011); Slatnar, Klancar, Stampar, & Veberic (2011) and Badii, Farahnaky, & Behmadi (2014) have recently carried out some research on fig fruit. Fig extract, however, does not have a long shelf life. Therefore, it needs to be concentrated, or converted to a powder with longer‐lasting storage characteristics. Drying is the major food processing operation which can enhance the shelf life. Spray drying has a short‐timed process and operational conditions which can be maintained under control. It is broadly used to produce high‐quality powders regarding water activity, color, flavor, and nutritional values from various foods and fruit and vegetable juices/extracts (Abadio, Domingues, Borges, & Oliveira, 2004; Daza et al., 2016; Quek, Chok, & Swedlund, 2007; Tonon, Brabet, & Hubinger, 2008). Spray drying of fig extract can be used for the production of healthy foods because it is considered as some kind of encapsulation (Encina, Vergara, Giménez, Oyarzún‐Ampuero, & Robert, 2016). Spray drying process is very effective in preparing any easy‐to‐use and log‐lasting powder which, after reconstitution, is more or less similar in properties to the original juice, commonly known to be directly in relation to drying conditions. Goula and Adamopoulos (2005); Quek et al. (2007); Tonon et al. (2008); Fazaeli, Emam‐Djomeh, Kalbasi‐Ashtari, and Omid (2012); Zareifard, Niakousari, Shokrollahi, and Javadian (2012) have published some useful reports regarding the operating conditions and qualitative characteristics of spray‐dried juice/extract powders (Fazaeli et al., 2012; Goula & Adamopoulos, 2005; Quek et al., 2007; Tonon et al., 2008; Zareifard et al., 2012).

Iran has produced 87, 522 tons of the fig fruit in 2006 and among fig‐producing countries. The country ranks third worldwide. In terms of production and exportation of fig, Iran along with Turkey, Egypt, Greece, Algeria, and Morocco, account for 70 percent of the global annual fig production. The *Sabz* cultivar belonging to the Smyrna type figs is the most common exportable Iranian local fig among other varieties, and in Fars province particularly Estahban district, estimated to be the most important producers of *sabz* fig in the country (Razavi et al., 2010). Although fig cultivation is widespread in Iran, large amounts of the fruit crop go to waste every year. Large portions of the figs are also damaged after falling on the ground and used as livestock feed. Meanwhile, they can be used in the production of fig extract and fig powder as a suitable ingredient for formulation purposes (Chang et al., 2016; Razavi et al., 2010).

Due to existing challenges and lack of documents in the field of fig syrup or powder production, as well as presence of strong demand for turning waste to high value products, this study examined the production of fig extract from waste figs under mild extraction conditions. In the second step, fig extract was used as feed to the spray dryer, for the first time in the scientific literature, to prepare a powder with suitable physico‐chemical properties for the production of seedless fig paste or drink. Furthermore, the effects of drying aids and spray drying operating conditions were investigated on the quality of the obtained powder.

## 2 MATERIALS AND METHOD

### 2.1 Materials

Maltodextrin (MD) (DE = 12, Tongaat Hulett Starch, South Africa) and low‐methoxylated pectin (LMP) (Belgium) were obtained from food ingredient suppliers and companies. Fig samples (*Ficus carica* L. cv. *Sabz*) were purchased from Estahban District, Fars Province, Iran. Approximate composition of fig samples including moisture content (8.4%), protein (4.4%), total fiber (11.5%), lipid (2.2%), Polysaccharide (70.5%), and ash (3%) were measured according to the AOAC (2000) methods.

### 2.2 Preparation of fig extract

In order to optimize extraction conditions, operations were accomplished at three different temperatures (5, 25, and 40°C). Fig to water ratios were (1:1.0, 1:1.5, and 1:2.0 w/w) and storage times were 12, 24, 48, and 72 hr. Fig fruits were crushed into smaller pieces, and then they were soaked in water and were kept in the static mode for various durations as stated above. According to our preliminary results, the fig extract was produced without heating because it could divide the extract to liquid part at the top and solid part at the bottom and furthermore, it could make it dark. The optimum extraction condition of fig extract was provided by a temperature of 5°C, a fig to water ratio of 1:1.5 W/w, and extraction time of 72 hr which produced fig extract with 14% ± 0.5°Brix. The mixture was refrigerated during the soaking process, until total soluble solid (TSS) reached 14 ± 0.5% in the liquid phase. The extract was then passed through a sieve (with a mesh size of 400 μ), so that large suspended solids were removed from the liquid. In the 1:1.0 ratio of fig to water (w/w), figs absorbed water completely and no fig extract was produced. Fig extract spoiled in temperature of 25 and 40°C, however, no growth occurred at 5°C. In addition, after 72 hr, 24 hr, and 12 hr at the extraction temperature of 5°C, 25°C, and 40°C, respectively, the fig extracts achieved a constant maximum °Brix of 22. Other conditions either led to a low º Brix value or caused the extract to be spoiled.

### 2.3 Analysis of the fig extract

The total soluble solid (14.0% W/W ± 0.3) content of the fig extracts was determined through a refractometer. Total solids measurements (15.34% W/W ± 0.04) were done according to the AOAC method (Jumah, Tashtoush, Shaker, & Zraiy, 2000). The pH value (4.88 ± 0.12) was determined using a pH meter. The titratable acidity (0.30% W/W ± 0.02) was measured based on the main acid of fig fruit concentration and citric acid (C6H8O7) (Horwitz, Chichilo, & Reynolds, 1970). The concentrations of ash (0.67% W/W ± 0.09), glucose (3.7% W/W ± 0.2), and fructose (2.8% W/W ± 0.1) were determined according to Amador method (Amador, 2008). Protein contents (0.28% W/W ± 0.04) and fat contents (0.09% W/W ± 0.03) of the fig extract were determined using AOAC methods (Latimer, 2012). It should be noted that there are several varieties of figs and it is expected that the characteristics of its products would be different. Furthermore, different extraction methods affect fig extract properties.

### 2.4 Glass transition temperature

The glass transition temperature (T_g_) of the fig extract was measured using a differential scanning calorimeter (DSC) (DSC1‐METTLER TOLEDO, Switzerland). The DSC was calibrated using indium *(*T_m,onset_ = 156.6, ∆H = 28.45 J/g) and at empty sealed pan was considered as the reference. A quantity of 5 mg of samples were embedded within the aluminum pans and sealed hermetically and heated from −50 to 200 at a rate of 10/min. Onset, inflection and end T_g_ were obtained from the graphs using the STAR^e^ system software. All the analyses were conducted in triplicate. The extract was refrigerated so that it can be used for either further processing or feeding the spray dryer.

### 2.5 Spray drying conditions

Spray drying is a technique whose outcome relies on the feed characteristics and drying conditions and settings. The combination of these effects can change various product parameters such as final moisture content, particle size, density, etc. The findings also revealed a positive relationship between T_o_ and Qa. Our results highly correlated with some of the previous studies (Goula & Adamopoulos, 2005; Zareifard et al., 2012). Small variations in T_o_ were observed with various ratios of MD: LMP (70:0, 56:14, and 49:21 g per 1 kg of extract feed to the dryer), but no apparent trend was detected.

It can bestated that for a given T_i_ and feed rate, at a higher air flow rate, the specific enthalpy of the inlet air increases, the residence time decreases, and as a result, the T_o_ increases. It is known that the rate of heat transfer exceeds the rate of mass transfer during the falling rate period. This aspect was not examined and the compressed air pressure through the nozzle was kept constant at 1.5 bars. It is therefore assumed that variations in the compressed air flow rate through the nozzle also affects T_o_. As the compressed air flow rate increases, the droplets become smaller and this results in a larger surface area that can increase heat transfer rate. It is well known that T_o_, as an important parameter in the spray drying process, greatly affects many physico‐chemical and sensory characteristics of the product, and therefore, the researchers need to monitor it closely to achieve successful spray drying (Masters, 1991).

### 2.6 Physico‐chemical and reconstitution properties of powders

#### 2.6.1 Residual moisture content

Residual moisture content values (MC) are a crucial parameter which can influence many properties of hygroscopic powders, particularly the caking phenomena. The outlet temperature of air is the determining parameter for lower moisture content values in products which are spray dried (Saravacos, 1967).

The MC of the powders was always less than 6% regardless of the MD:LMP ratio, the Qa, or the Ti. Hence, powders with low moisture content which were obtained throughout various runs in this Study could be considered as indicators of a successful drying process. By an increase in the inlet air temperature, powders with lower moisture content were achieved. The reason was because of the fact that by increasing the inlet temperature of air, the heat transfer rate to the extract particles/droplets increases as well which later on produces a greater driving force for the evaporation of moisture and also results in powders of lower moisture content (Quek et al., 2007). Furthermore, increasing the inlet air temperature reduces relative humidity of air, resulting in a higher capacity for moisture intake (Masters, 1991). On the contrary, increasing the air flow rate usually results in powders with higher moisture content. This may be because of the shorter staying of particles/droplets in the chamber and smaller values of vapor pressure of water (Goula & Adamopoulos, 2005).

Examination of the optimization trials proved that measurable production of powders with less than 70 g of MD and LMP mixture as drying aid in 100 g of the feed prior to the spray drying was almost impossible. The extracts either did not dry or were too sticky to exit the chamber. The condition could also be that the cyclones were at lower temperatures. Increasing the concentration of the drying aid to 60% did not accelerate the powder production process. Therefore, the level of drying aid was set at 50% of TSS in the extract. An increase in maltodextrin from 49 g to 70 g in the MD‐LMP ratio helped in obtaining a powder with a moisture content of about 5% to 6% at 150°, 480 m^3^/hr. This variation, regarding the investigated variables however, did not have a statistically significant effect (*p < .05*) on the MC of the final dried product. Jittanit, Niti‐Att, and Techanuntachaikul (2010) showed that the moisture contents of pineapple juice powders were between 4.0% and 5.8%. An increase in the drying temperature and the maltodextrin content led to a powder with lower moisture content (Jittanit et al., 2010). Tuyen, Nguyen, and Roach (2010) reported that by increasing the maltodextrin concentration of the drying aid from 10% to 20%, the MC of Gac (*Momordica cochinchinensis*) fruit powder reduced from 4.87% to 4.06% (Tuyen et al., 2010). Similar results have been reported for spray dried tomato powder (Goula & Adamopoulos, 2005) and lime juice powder (Zareifard et al., 2012).

#### 2.6.2 Bulk, particle, and tapped densities

**Figure 1:**
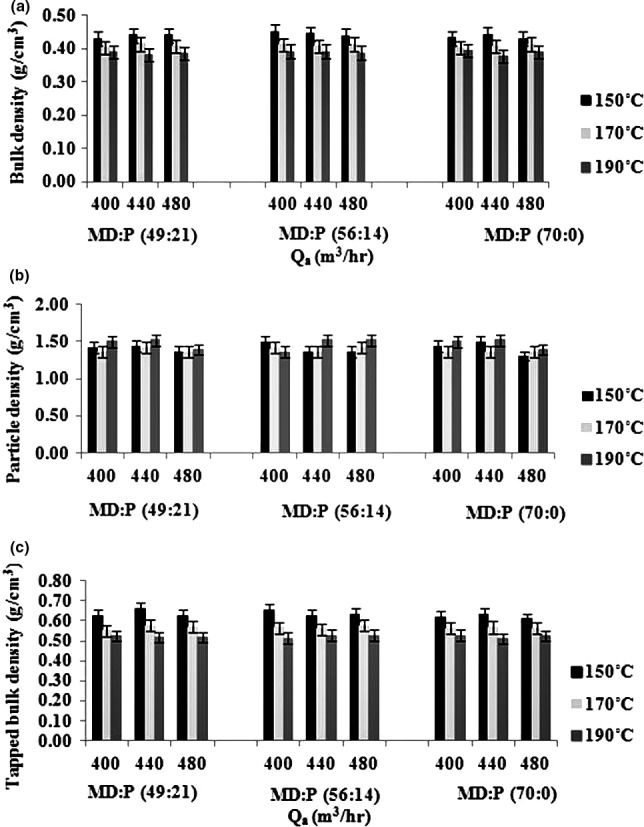
The effects of various drying aids, inlet air temperatures, and air flow rates on bulk density (a), particle density (b) and tapped density of the powders (c), (data represent means ± standard deviations from triplicate analysis)

Particle size and its distribution are two determining factors in food powder's bulk properties (Ahmed & Ramaswamy, 2006; Koç, Sakin‐Yılmazer, Kaymak‐Ertekin, & Balkır, 2014). Bulk density (BD) of the drying aid mixture was about 3.20 g/cm^3^. BD of the powders varied from 0.38 to 0.45 g/cm^3^ (Figure 1a) which was not statistically significant (*p* < 0/05), however, and an increase in the inlet air temperature from 150°C to 190°C led to a 20% decrease in BD, regardless of the air flow rate and the MD to LMP ratio. The drop in BD may be due to a faster evaporation rate at a higher temperature which, in turn, may result in lower shrinkage of the droplet and, therefore, lead to a product with a more porous structure. It has been reported that an increase at the concentration of feed, temperature, and flow rate, the results lead to a decreased T_o_ and powder bulk density (Jumah et al., 2000; Masters, 1991).

When Ti is constant, by an increase in the air flow rate, a 10% drop might be observed in BD for all the MD:LMP ratios. Generally, the air flow rate did not have any significant effect (*p < *.05) on the BD of the powders and no significant difference (*p* < 0/05) was observed between the powders in this regard. The small decrease in the BD, which was due to an increase in the air flow rate, may be attributed to the production of powders with higher residual moisture. By having higher powder moisture content, more particles tend to stick together, thereby leaving more distances between them which produce a product with larger bulk volume. These findings largely correlate with the findings of Zareifard et al., (2011); Goula and Adamopoulos (2005). Many researchers have reported the presence of a higher concentration of the drying aid in the feed to the spray dryer, which may greatly reduce BD. Nadeem, Torun, and Özdemir (2011), however, observed that an increase in the drying aid concentration in the feed when producing mountain tea powder by means of spray drying led to an almost 13% increase in the BD (Nadeem et al., 2011). Shrestha, Ua‐Arak, Adhikari, Howes, and Bhandari (2007) observed that increasing MD concentration of the orange juice powder resulted in a decrease in the BD (Shrestha et al., 2007). In this study, only one concentration of the drying aid, that is, 50% of the TSSC, was used in the feed in all the 27 runs. One must, therefore, be cautious about drawing any conclusion with regard to the effect of various concentrations of the drying aid in the feed on the BD. Within the scope of this study, however, an increase in MD concentration led to a decrease in BD with all the air flow rates and inlet air temperatures. Results of statistical analysis revealed that variation in MD concentration in the MD:LMP ratio had a statistically significant effect (*p < *.05) on BD and led to a decrease in it. The reason may be possibly because of the lower moisture content of the products or the higher volume of the trapped air, since MD is a skin‐forming material (Fazaeli et al., 2012).

**Figure 2:**
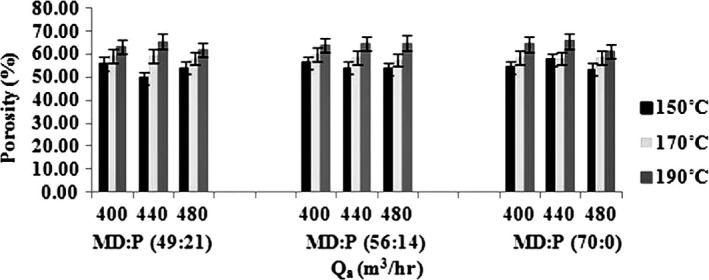
The effects of various drying aids, inlet air temperatures, and air flow rates on porosity of the powders (data represent means ± standard deviations from triplicate analysis)

The density of powders in most food powders is something between 1.40 and 1.60 g/cm^3^ if they are not rich in salt or fat (Barbosa‐ Cánovas & Juliano, 2005; Koç et al., 2014). The particle density of the powders varied from 1.31 to 1.52 g/cm^3^ (Figure 2b). The results indicated that variation in MD in the MD:LMP ratio, the air flow rate, and the inlet air temperature did not significantly affect *(p < *0/05*)* the particle density of the powders.

As Figure 2c shows the tapped bulk density (TBD) for the fig extract powders ranged from 0.5 to 0.7 g/cm^3^. Increasing the inlet air temperature resulted in a small decrease in the TBD. There were significant variations (*P < *0/05) in the TBD as a result of changes in the MD:LMP ratio which were not statistically significant (*p < *0/05). Increasing the air flow rate, however, for all the inlet temperatures and the MD:LMP ratios did not have a statistically significant effect (*p < *0/05) on the TBD. The difference between bulk density (0.38–0.45 g/cm^3^) and tapped density (0.51–0.65 g/cm^3^) indicates packing and flow behavior of particulate materials and smaller differences indicate a better flow and a less stable packed system (Couto et al., 2011).

#### 2.6.3 Porosity

Porosity is a parameter usually important in rehydration of dried products and controlling rewetting speed as well as appearance and flavor release. Porosity of the produced powders varied from 49% to 65%, depending on the inlet air temperature, the air flow rate, and the MD:LMP ratio (Figure 2). With all the air flow rates and MD:LMP ratios, increasing the inlet air temperature led to an increase in the porosity as well. This is largely because of a lower moisture content at higher temperatures which, in turn, may lead to less compression and, therefore, higher of porosity of the powders (Saravacos, 1967). Similarly, increasing the air flow rate resulted in an increase in porosity, but to a much lesser extent. During the drying and transport processes, particle‐particle abrasion is one other reason for low porosity of Spray‐dried powders at higher temperatures and higher air flow rates (Jangam & Thorat, 2010). The variation in MD in the MD:LMP ratio, however, did not have any statistically significant effect (*p < *.05) on porosity. Caparino et al. (2012), in their study on the effect of drying methods on the physical properties and microstructures of mango, found that with an increase in the MD concentration, porosity increased as well (Caparino et al., 2012). Goula, Karapantsios, Achilias, and Adamopoulos (2008) observed that an increase in MD caused a larger amount of air to be trapped within the particles and led to less dense and highly porous powders (Goula et al., 2008).

#### 2.6.4 Dispersibility

**Figure 3:**
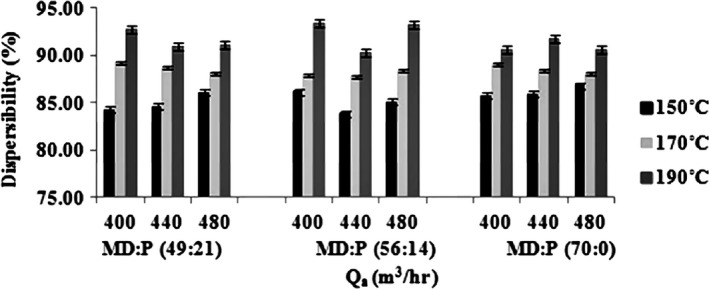
The effects of various drying aids, inlet air temperatures, and air flow rates on dispersibility of the powders (data represent means ± standard deviations from triplicate analysis)

Size is one of the factors affecting dispersion of a particle. Fig extract particles produced during the spray drying process had a dispersibility range of about 83% to 94% (Figure 3). Increasing the inlet air temperature led to a statistically significant (*p < *0/05) increase in the powder dispersibility at all MD:LMP ratio and air inlet temperature. However, the effect of air flow rate on porosity of the powders was less than that of inlet air temperatures. The dispersibility of the powders decreased by increasing the MD in the MD:LMP ratio but this was not statistically significant (*p < *0/05). Better powder dispersibility at higher inlet air temperatures may be due to the larger‐sized and less moist particles formed at these temperatures. A higher amount of residual moisture in a powder can usually result in a powder with higher stickiness and, as a result, a powder with a lower dispersion. Furthermore, powders with high concentrations of low molecular weight sugars, for example, fig extract powder, tend to absorb moisture quickly. Therefore, in the juice, sugar crystallization can begin and bridges of solid crystal between the particles can be formed which can contribute to the reduced dispersibility of the powders (Jinapong, Suphantharika, & Jamnong, 2008).

#### 2.6.5 Particle size distribution

**Figure 4:**
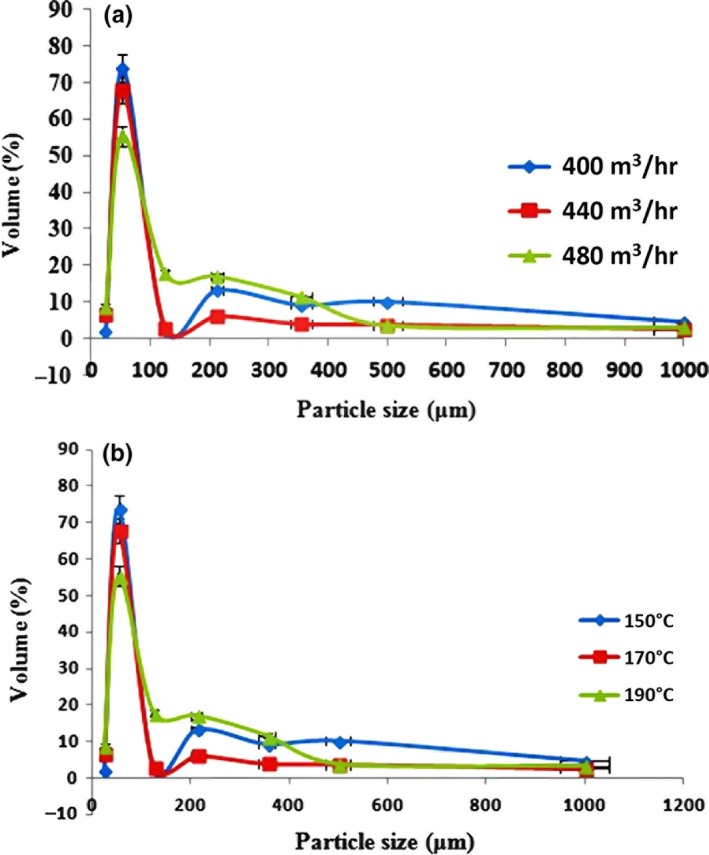
Particle size distribution of powders with different air flow rates, a temperature of 170°C, and an MD:P ratio of 49:21(a), particle size distribution of powders with different temperatures, an air flow rate of 400 m^3^/hr, and an MD:P ratio of 49:21 (b), (data represent means ± standard deviations from triplicate analysis)

Being an important characteristic of food powders, particle size distribution can affect many of food powders' properties, such as viscosity, density, porosity, chemical reactivity, electrical and thermal conductivity, color, and appearance (Johnson, Anantheswaran, & Law, 1996; Shittu & Lawal, 2007). Figure 4a and b show the variations in particle size distribution of powders produced through using the MD:LMP ratio of 49:21, as described above, and various air flow rates (400–480 m^3^/hr) and inlet air temperatures (150–190°C).

Particle size distribution may be influenced by various processing parameters and feed characteristics. In this study, more than 70% of the produced powders were about 53 μ. An increase in the inlet air temperature led to a significant increase (*p < *0/05) in the particle size distribution of the powder. By an increase in the inlet air temperature, particles became larger‐sized because of the greater swelling of particles (Tonon et al., 2008). When the inlet air temperature is higher, the drying rate will be increased as well. Because of this, larger‐sized particles are produced, due to the early formation of a structure, and would prevent them from shrinking during the drying process (Tonon et al., 2008). The air flow rate had a statistically significant effect (*p < *0/05) on the particle size distribution as well. However, it seems that variations in concentrations of the drying components had no statistically significant effects (*p < *0/05) on the particle size distribution of powders. The highest particle size distribution was obtained at the MD:LMP ratio of 49:21.

#### 2.6.6 SEM images

**Figure 5:**
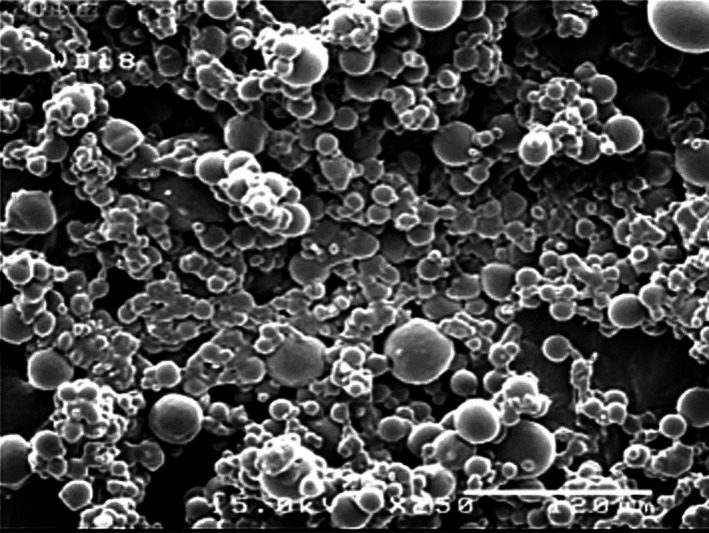
Scanning electron micrographs of fig extract powders with a MD:P ratio of 49:21, an air flow rate of 400 m^3^/h and a temperature of 170°C (Magnification 250 × ; scale bar=12 μm)

Figure 5 shows the SEM image for the fig extract powder at the air flow rate, inlet air temperature, and MD:LMP ratio of 400 m^3^/hr, 170°C and 49:21, (optimum drying condition), respectively. An overall observation of the image indicates that the most particles are spherical, ranging in size from 5 to 50 μ. Increasing the distribution of particle size often results in the rapid formation of a dried layer on the droplet surface. The increase in the size of the particles was due to case hardening of the droplets at the higher temperatures which resulted in the formation of Vapor‐impermeable films on the droplet surfaces which is, in turn, followed by vapor bubble formation and droplet expansion. In addition, the production of higher moisture content would tend to have a higher bulking weight, caused by the presence of water, which is very denser than the dry solid. This hardened skin prevents the moisture from exiting out of the droplet, and therefore, the particle size increases. Chegini and Ghobadian (2007); Phoungchandang and Sertwasana (2010) found similar results to our study, they reported that the particle size of ginger powder ranged from 47 to 84 μm and increased with an increase in the inlet air temperature from 120 to 150°C, but decreased with an increase at the concentrations of maltodextrin and liquid glucose from 0 to 10% (Chegini & Ghobadian, 2007; Phoungchandang & Sertwasana, 2010).

#### 2.6.7 Color analysis

The variation in color parameters of the powders: L*, a*, and b*, with the inlet air temperatures, air flow rates, and MD:LMP ratios are shown in Tables 1. Findings show that the inlet air temperatures, air flow rates, and drying aids had no statistically significant effect (*p < *0/05) on the L* (lightness) and a* (greenness) values**.** The rise in the inlet air temperatures, however, significantly decreased (*p < *0/05) the b* (yellowness) value. On the other hand, variations in the air flow rate and MD:LMP ratio had no statistically significant effects (*p < *0/05) on the b* value. Higher inlet air temperatures tainted the color of the dried product because they caused rapid pigment oxidation and nonenzymatic browning reactions. Generally, operating the spray drier with a higher inlet air temperature, because of the thermal degradation of pigments, results in a greater loss of yellow color. In their studies, Sousa, Borges, Magalhães, Ricardo, and Azevedo (2008) and Tuyen et al. (2010) found that increasing the inlet air temperature decreased the a* and b* values of tomato and Gac fruit aril powders, respectively. Other studies have also indicated the significant influence (*p < *0/05) of air temperature on color variation in carrot powder (Chen, Peng, & Chen, 1995) and tomato products (Chen et al., 1995; Shi, Le Maguer, Kakuda, Liptay, & Niekamp, 1999; Sousa et al., 2008; Tuyen et al., 2010).

**Table 1:** Colorimetric analysis and glass transition temperatures (T_g_) of the best produced fig extract

T_g_			Colorimetric analysis based on Hunter Lab factors		
Onset point (°C)	Inflection point (°C)	End point (°C)	L*	a*	b*
−12.17 ± 0.07	−5.11 ± 0.03	6.15 ± 0.04	−44.66 ± 1.02	−3.33 ± 0.46	28.33 ± 0.12

#### 2.6.8 Sensory evaluation of the cakes

Table 2 describes the organoleptic characteristics of two cakes; one made with fig extract and the other made without fig extract. In this regard, color, texture, mouth feel, flavor, sweetness, and overall acceptability of the samples were surveyed. The results showed that the cake made with fig extract obtained significant (*p < *.05) higher scores for color and flavor acceptability. Overall, no statistically significant difference (*p < *0/05) was observed between the two cakes samples in the remaining characteristics.

**Table 2:** Sensory Evaluation of the two cakes (one made with fig extract and the other made without it)

Sampels	Colour	flavor	Taste	Texture	Mouth feel	Total score
Control (Cake made without fig extract)	3.14 ± 0.03^b^ *	3.12 ± 0.05^b^	3.11 ± 0.07^a^	3.00 ± 0.14^a^	3.27 ± 0.13^a^	3.43 ± 0.07^b^
Cake made with fig extract	4.15 ± 0.09^a^	4.11 ± 0.12^a^	3.12 ± 0.02^a^	3.26 ± 0.11^a^	3.56 ± 0.10^a^	5.20 ± 0.13^a^

*In each column different superscript letters indicate significant differences (*p *<* *.05).

## 3 CONCLUSIONS

To conduct this study, the optimal extraction conditions for producing fig extract is suggested to be 5°C at 72 hr while the fig to water ratio of 1:1.5 is found to be most appropriate. These result in minimum heat damage and nutrient loss. Fig extract powder was produced from the natural fig extract through using a pilot scale two‐fluid nozzle spray dryer. The study then investigated the effects of drying operating conditions and applying a variation in components involved in drying, that is, the ratio of maltodextrin to pectin, and how this can affect the physico‐chemical properties of the produced powder. Based on the findings of this study, an increase in the inlet air temperature led to a decrease in powder moisture content, bulk density and degree of yellowness of the powders. Furthermore, increasing the inlet air temperature led to an increase in porosity, dispersibility, and particle size distribution. In addition, an increase in the air flow rate resulted in an increase in moisture content, dispersibility and porosity of the powders. The effect of variation in the maltodextrin to pectin ratio on the powder was only statistically significant (*p < *.01) regarding the moisture content, bulk density, and porosity. In conclusion, by considering the relevant variables investigated in this study, the best powder was produced at a temperature of 170°C using an air flow rate of 400 m^3^/h and a MD:LMP ratio of 79:21. Our results suggest that producing fig powder under the mentioned conditions can be a good strategy for converting waste figs to high value products capable of being used in the formulation of healthy food products.

## CONFLICT OF INTEREST

Any of authors have no conflict of interest to declare.
